# Hen Egg White Lysozyme (HEWL) Confers Resistance to Verticillium Wilt in Cotton by Inhibiting the Spread of Fungus and Generating ROS Burst

**DOI:** 10.3390/ijms242417164

**Published:** 2023-12-05

**Authors:** Wenfang Guo, Gangqiang Li, Nan Wang, Caifeng Yang, Huakang Peng, Mengqi Wang, Dehu Liu

**Affiliations:** Biotechnology Research Institute, Chinese Academy of Agricultural Sciences, Beijing 100081, China

**Keywords:** Hen egg white lysozyme, antifungal protein, transgenic cotton, disease resistance, Verticillium wilt, ROS burst

## Abstract

Verticillium wilt is a soil-borne vascular disease caused by the fungal pathogen *Verticillium dahliae*. It causes great harm to upland cotton (*Gossypium hirsutum*) yield and quality. A previous study has shown that Hen egg white lysozyme (HEWL) exerts strong inhibitory activity against *V. dahliae* in vitro. In the current study, we introduced the *HEWL* gene into cotton through the *Agrobacterium*-mediated transformation, and the exogenous HEWL protein was successfully expressed in cotton. Our study revealed that HEWL was able to significantly inhibit the proliferation of *V. dahlia* in cotton. Consequently, the overexpression of HEWL effectively improved the resistance to Verticillium wilt in transgenic cotton. In addition, ROS accumulation and NO content increased rapidly after the *V. dahliae* inoculation of plant leaves overexpressing HEWL. In addition, the expression of the PR genes was significantly up-regulated. Taken together, our results suggest that HEWL significantly improves resistance to Verticillium wilt by inhibiting the growth of pathogenic fungus, triggering ROS burst, and activating PR genes expression in cotton.

## 1. Introduction

Cotton disease is one of the most important factors causing the decline in cotton yield and quality. In particular, Verticillium wilt results in a severe yield reduction in cotton. In China, the primary pathogen causing the Verticillium wilt of cotton is *V. dahliae*, which can also infect about 660 plant species belonging to 38 families, including *Solanaceae*, *Fabaceae*, *Rosaceae*, *Brassicaceae* and *Asteraceae* [1,2]. In the beginning, *V. dahliae* derived from the soil starts colonizing cotton roots and enters the vascular structure. The vascular system produces a large number of mycelia and conidia which proliferate and spread to other plant parts. Ultimately, it damages the plants by blocking the vascular system and releasing toxic proteins [2,3]. However, the complete infestation progress is not completely understood to date. The vascular tissues of plants can transmit pathogens, although this is not the only way [2]. Verticillium wilt is a typical vascular disease that can affect crops for a long time and is extremely difficult to cure once infected [4]. Currently, no highly disease-resistant cotton varieties are available in China as resources. Therefore, improving cotton varieties to increase disease resistance and reduce the impact of Verticillium wilt on cotton yield is a significant challenge in the cotton breeding industry.

Biological breeding is an essential technology for the rapid and directional breeding of new crop varieties. *Bacillus thuringiensis* (Bt) cotton has been successfully commercialized widely for many years, and it effectively resolves the threat of bollworm infestation to cotton yield [5]. In recent years, several studies have shown that the overexpression of foreign genes in cotton can improve the resistance to Verticillium wilt to some extent. The ectopic expression of plant defensin NaD1 from *Nicotiana alata*, harpin protein Hpa1Xoo from *Xanthomonas oryzae*, and cell surface-localized immune receptor Ve1 from *Solanum lycopersicum* show enhanced tolerance to Verticillium wilt in cotton [6,7,8]. A fusion protein of the antifungal peptide BbAFP1 and the ergosterol domain of ergosterol targeting the fungal cell wall and cell membrane are expressed in cotton, which show higher disease resistance compared to the expression of BbAFP1 alone [9]. In addition, the overexpression of endogenous genes derived from cotton can also improve its resistance to Verticillium wilt. Overexpression of *GhMYB4* (MYB transcription factor), *GhPFN2* (profilin gene), *GhDIR1* (dirigent1 gene), and *GbRLK* (receptor-like kinase gene) in cotton improve the defense response of plants to pathogens [10,11,12].

The plant defense responses mainly include constitutive defense system, inducible defense (PAMP-triggered immunity, PTI, and effector-triggered immunity, ETI), and systemic-acquired resistance (SAR) [13,14,15,16]. Salicylic acid (SA) and jasmonic acid (JA) are important signal molecules in the SAR signal pathway. *Gbvdr6* from the Verticillium wilt-resistant cotton Hai7124 and *NPR1* from *Arabidopsis* confer resistance to Verticillium wilt in cotton through the regulation of the JA/ET and SA signaling pathways [17,18]. Previously, many cotton resistance-related genes have been identified. For instance, plant cell wall-associated receptor-like kinases *GhWAKL*, pectin methylesterase-inhibiting protein *GhPMEI3*, ribosomal protein *GhRPS6*, and laccase gene *GhLAC15* are shown to play essential roles in cotton resistance to pathogen infection [19,20,21,22]. During *V. dahliae* infection in cotton plants, several transcription factors are also involved in regulating plant disease resistance. BEL1-Like TF *GhBLH7-D06* and HD-ZIP I TF *GhHB12* negatively regulate cotton resistance to Verticillium wilt [23,24], while R2R3-type MYB TF *GhODO1* and WRKY TF *GhWRKY1-like* are known to positively regulate cotton resistance to Verticillium wilt [25,26].

Lysozyme is highly abundant in egg white, accounting for about 3–4% of the total protein content [27]. Hen egg white lysozyme (HEWL) is a c-type lysozyme that hydrolyzes β-1,4 glycosidic bonds between the N-acetylmuramic acid (NAM) and N-acetyl glucosamine (NAG) of peptidoglycan in the bacterial cell wall [28]. Further, some studies have also shown the antifungal activity of HEWL. Our previous study indicated that HEWL could effectively inhibit the growth of fungal pathogens, *V. dahliae* and *F. oxysporum*, and that the antifungal rate was as high as 75.7% compared with phosphate buffer (pH6.2) [29]. In this study, the *HEWL* gene driven by the plastocyanin promoter (*PetE* Pro) was transformed into *G. hirsutum* R15 using the *Agrobacterium*-mediated method. We observed that *HEWL* confers resistance to Verticillium wilt in cotton under greenhouse and field conditions. *HEWL* transgenic cotton exhibited ROS burst and increased the expression of pathogenesis-related (PR) genes expression under biotic stress. The *V. dahliae* biomass was also lower in transgenic cotton than in wild-type cotton. Altogether, our results suggested that that constitutive expression of *HEWL* in cotton could increase resistance to Verticillium wilt.

## 2. Results

### 2.1. Generation of HEWL Transgenic Cotton Plants

**Figure 1:**
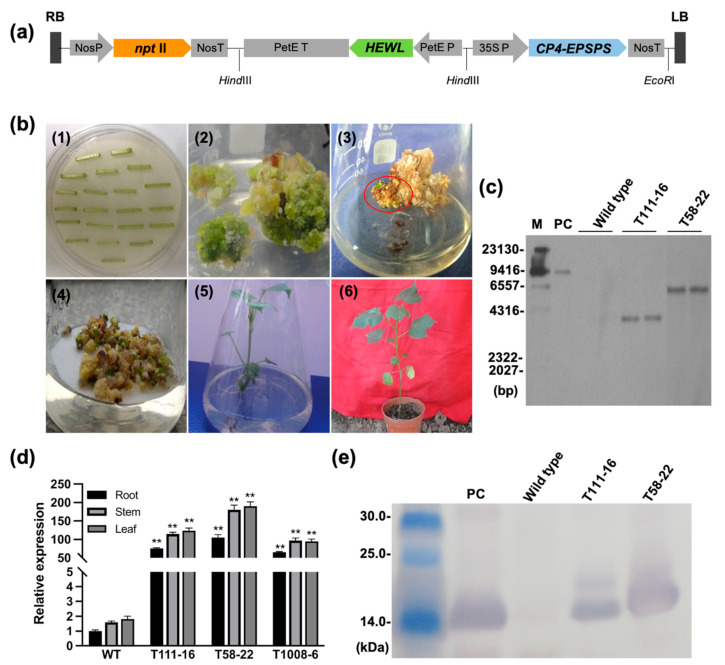
Generation of transgenic cotton plants overexpressing *HEWL.* (**a**) Schematic diagram of plant expression vector pBI121-35S::*CP4_EPSPS*-PetE::*HEWL*. NosP: nopaline synthase gene promoter; NosT: nopaline synthase gene terminator; PetE P: plastocyanin gene promoter region and 5′ UTR from *Medicago sativa*; PetE T: plastocyanin gene terminator region and 3′ UTR; 35S P: Cauliflower mosaic virus 35S promoter; LB, left border; RB, right border. (**b**) Genetic transformation of cotton: 1. Co-culture of explants with *Agrobacterium*. 2. Primary resistant callus. 3. Embryogenic callus. 4. Embryoids. 5. Regenerated plants. 6. Grafted T_0_ generation plants. (**c**) Southern blot analysis of *HEWL* transgenic plants. M: DNA molecular-weight marker. PC: the plasmid with *Not*I as a positive control. (**d**) qRT-PCR analysis of expression levels of *HEWL* in roots, stems, and leaves of three homozygous lines. (**e**) Western blot analysis of HEWL proteins in transgenic cotton homozygous lines. β-actin: cotton reference protein as a control. T58-22, T111-16, and T1008-6: transgenic cotton homozygous lines; WT (wild type): non-transgenic cotton plant R15; PC: HEWL from egg white as a positive control. Data were obtained from three independent experiments and represent the means ± SE; ** *p* < 0.01.

The plant expression vector pBI121-35S::*CP4_EPSPS*-PetE::*HEWL* was shown in Figure 1a. PetE promoter from plastocyanin gene 5′ UTR of *Medicago sativa* was used upstream of *HEWL* ORF. Moreover, the expression of glyphosate-resistant gene *CP4-EPSPS* as a selectable marker gene in the vector was essential in selecting transgenic plants. The plant expression vector was introduced into upland cotton R15 via the *Agrobacterium*-mediated transformation method. The process of cotton genetic transformation went through co-cultivation formation, the generation of primary callus, embryogenic callus, embryoid body formation, and finally differentiation into regenerated plants, which were grafted into 25-day Hai 7124 (Figure 1b). The exogenous genes *CP4-EPSPS* and *HEWL* were identified by PCR in the regenerated cotton plants (Figure S2). Three homozygous lines T111-16, T58-22, and T1008-6 were identified through three generations of continuous selfing, and the target genes were detected in all plants of homozygous transgenic lines which showed resistance to glyphosate (Figure S3). Southern blot analysis revealed a single copy insertion of the exogenous gene in lines T111-16 and T58-22 (Figure 1c). qPCR confirmed the high expression of *HEWL* in the roots, stems, and leaves of transgenic cotton, and the expression level of line T58-22 was higher than that of line T111-16 and T1008-6 (Figure 1d). Further, Western blot analysis confirmed the expression of HEWL protein in the transgenic cotton. The results showed that the expression level of HEWL in T58-22 was higher than that in line T111-16 (Figure 1e), which was consistent with the qPCR results.

### 2.2. HEWL Expression Improved Resistance to Verticillium Wilt in Transgenic Cotton

**Figure 2:**
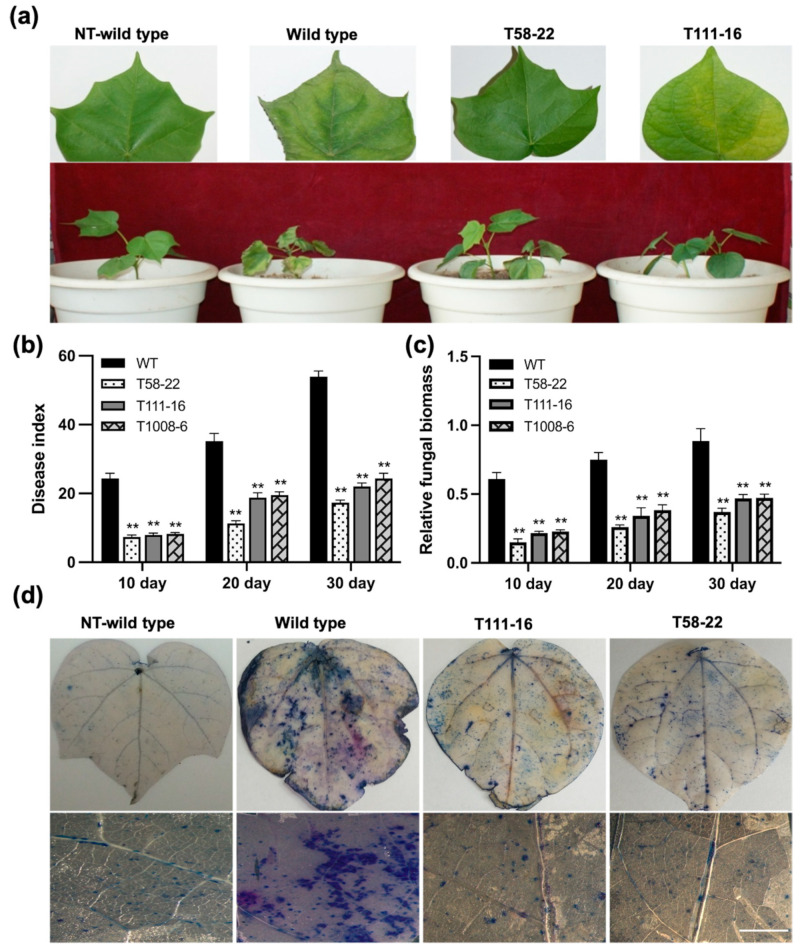
Resistance to *V. dahliae* of *HEWL* transgenic cotton homozygous lines in the greenhouse. (**a**) Resistance phenotypes of transgenic cotton lines T58-22 and T111-16 in the greenhouse. (**b**) DIs of transgenic cotton homozygous lines. (**c**) Relative fungal biomass of transgenic cotton homozygous lines. (**d**) Trypan blue staining of leaves from transgenic cotton homozygous lines plants after *V. dahliae* inoculation. Scale bars = 5 mm. T58-22, T111-16, and T1008-6: transgenic cotton homozygous lines; WT (wild type): non-transgenic cotton plant R15; NT-wild type: non-treatment wild type cotton plant. Data were obtained from three independent experiments and represent the means ± SE; ** *p* < 0.01.

We assessed the resistance of *HEWL* transgenic cotton to Verticillium wilt in the greenhouse. After inoculation with Vd991, transgenic cotton lines T58-22 and T111-16 that were growing normally and only had a small number of yellow spots, showed strong resistance to Verticillium wilt, while the leaves of wild-type cotton began to wither and fall off (Figure 2a). The disease indexes (DIs) of *HEWL* transgenic cotton lines were significantly lower than that of WT at 10 days, 20 days, and 30 days after inoculation with Vd991. The DI of the three lines was less than 30 and showed significant the resistance to the Vd991 strain (Figure 2b). The fungal biomass determination revealed that *HEWL* transgenic cotton lines was lower than fungal biomass in WT after inoculation (Figure 2c). Further, the trypan blue staining showed that *V. dahliae* caused the death of leaf cells in wild-type plants (more blue staining area), while the leaves of *HEWL* transgenic plants only had a smaller number of cells (Figure 2d).

**Figure 3:**
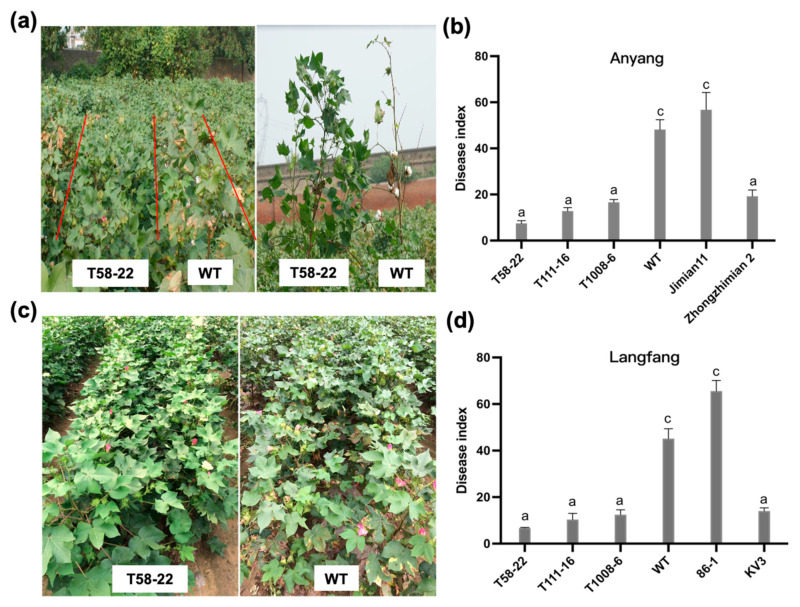
HEWL improved resistance to Verticillium wilt of transgenic cotton in *V. dahliae* disease-field. (**a**) Resistance phenotype of *HEWL* transgenic cotton in Anyang of Henan Province, China, in 2021. (**b**) The DIs of *HEWL* transgenic cotton in Anyang of Henan Province, China, in 2021. *G. hirsutum* cv. Zhongzhimain 2 as the resistance variety control; *G. hirsutum* cv. Jimian11 as susceptible variety control. (**c**) Resistance phenotype of *HEWL* transgenic cotton in Langfang of Hebei Province, China, in 2021. (**d**) The DIs of *HEWL* transgenic cotton in Langfang of Hebei Province, China, in 2021. *G. hirsutum* cv. Zhongzhimain KV3 as the resistance variety control; *G. hirsutum* cv. 86-1 as susceptible variety control. T58-22, T111-16, and T1008-6: transgenic cotton homozygous lines; WT: non-transgenic cotton plant R15. Data were obtained from four independent experiments and represent the means ± SE. The letters (a,c) indicate significant differences at *p* ≤ 0.01 according to a randomization one-way ANOVA test.

Further, we conducted field disease resistance experiments in Anyang, Henan Province, and Langfang, Hebei Province. In Anyang, we observed that most leaves of WT plants had light yellow patches on between leaf margins and leaf veins, which gradually expanded, and the leaves started to fall off the bottom of the plant (Figure 3a). The DI statistics in September revealed that the DI of *HEWL* transgenic cotton was significantly different from that of WT and the susceptible control “Jimian11”. In particular, the DIs of lines T58-22 and T111-16 were lower than 15, showing high disease resistance (HR) (Figure 3b). Also, *HEWL* transgenic cotton showed a better disease resistance phenotype (Figure 3c), and their DIs were all lower than 15, exhibiting high resistance in Langfang (Figure 3d). Altogether, these results indicated that the disease resistance was significantly improved in *HEWL* transgenic cotton.

### 2.3. HEWL Transgenic Cotton Enhances Disease Resistance by Generating ROS Burst and Activating PR Genes

**Figure 4:**
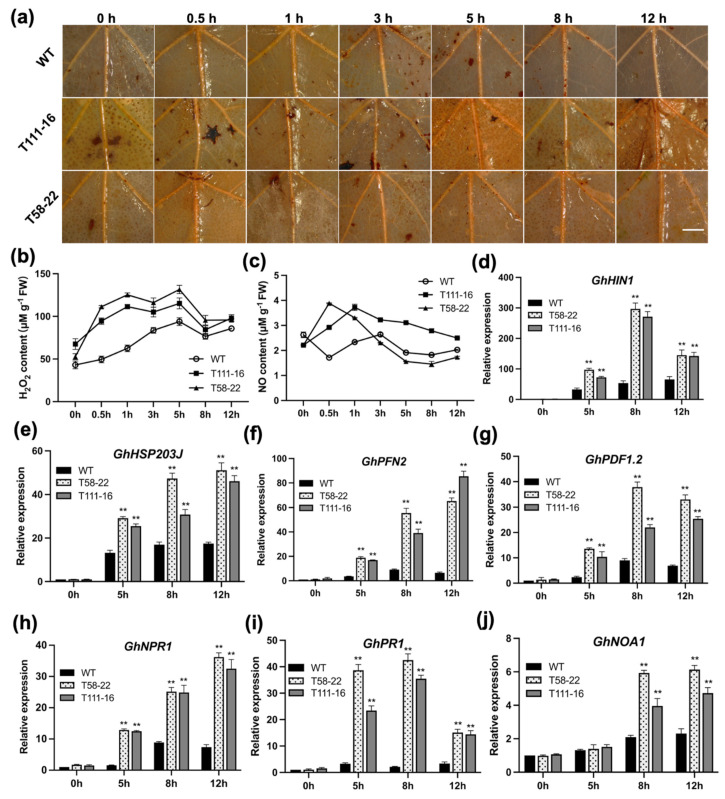
ROS burst in leaves of *HEWL* transgenic cotton dipped in a conidial suspension of *V. dahliae*. (**a**) In situ observation of ROS in *HEWL* transgenic cotton leaves with DAB staining dipped in a conidial suspension of *V. dahliae*. At least four independent leaves were used for this experiment. Scale bars = 5 mm. (**b**) H_2_O_2_ content of *HEWL* transgenic cotton leaves dipped in a conidial suspension of *V. dahliae*. (**c**) NO content of *HEWL* transgenic cotton leaves dipped in a conidial suspension of *V. dahliae*. (**d**–**j**) Relative expression of the PR genes of *HEWL* transgenic cotton dipped in a conidial suspension of *V. dahliae*. Data were obtained from four independent experiments and represent the means ± SE; ** *p* < 0.01.

In the current study, ROS burst was observed in *HEWL* transgenic cotton leaves infected with *V. dahliae*. We observed that the leaves of lines T58-22 and T111-16 rapidly showed reddish-brown precipitation after inoculation with *V. dahliae*, which was different from the phenotype of WT leaves, as detected by 3,3′-diaminobenzidine tetrahydrochloride (DAB) staining (Figure 4a). Further, we examined the H_2_O_2_ content in leaves after inoculation with *V. dahliae* and found that the H_2_O_2_ content of *HEWL* transgenic cotton was significantly higher than that of WT. In WT, the H_2_O_2_ content gradually increased and reached a peak at 5 h, while in *HEWL* transgenic cotton, it increased sharply with two peaks at 1 h and 5 h (Figure 4b). The NO content in *HEWL* transgenic cotton increased immediately and began to gradually decrease after inoculation with *V. dahliae* (Figure 4c). These results suggested that an immediate ROS burst was triggered in the transgenic cotton lines in response to biotic stress, activating hormonal signaling pathways.

HEWL was able to activate expression of PR genes in cotton. The charpin-induced 1 (*HIN1*) and *HSR203J* were the HR marker genes of hypersensitivity [30,31]. Previous studies demonstrated that the cotton profilin gene *GhPFN2* was involved in plant innate immune responses [32]. When plants were attacked by fungal pathogens, the *PDF1.2* gene encoding plant defensins was regulated by simultaneous jasmonate and ethylene signaling [33]. The *NPR1* (non-expressor of PR gene) gene was a key regulator of SA-mediated SAR and played an important role in plant basic defense [34]. Nitric oxide-associated protein 1 (NOA1) was involved in various abiotic stress responses and required for plants to resist pathogen infection [35]. After inoculation with *V. dahliae*, qPCR analysis revealed that the expression of *HIN1* and *HSR203J* was significantly up-regulated in the *HEWL* transgenic lines than the WT (Figure 4d,e). In addition, the PR genes *GhPFN2*, *GhPDF1.2*, *GhNPR1*, and *GhNOA1* were also significantly up-regulated in *HEWL* transgenic lines (Figure 4f–j). These results suggested that *HEWL* transgenic cotton responded to biotic stress by regulating the expression of PR genes.

### 2.4. HEWL Inhibits the Growth of V. dahliae in Cotton

**Figure 5:**
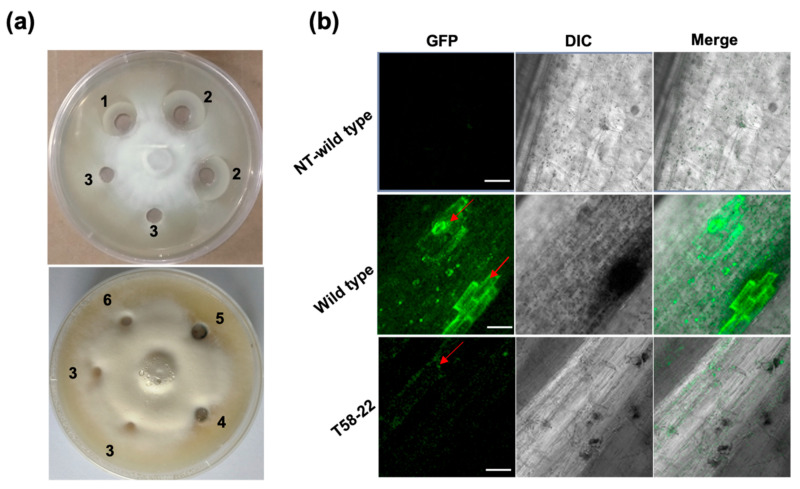
Overexpression of HEWL inhibited the spread of *V. dahliae* spores in transgenic cotton. (**a**) Antifungal activities of *HEWL* transgenic cotton against *V. dahliae* Vd991 on PDA plates. 1) 0.5 mg/mL HEWL from egg white; 2) 1 mg/mL HEWL from egg white; 3) ddH_2_O; 4) 1 mg/mL total protein from *HEWL* transgenic cotton line T111-16; 5) 1 mg/mL total protein from *HEWL* transgenic cotton line T58-22; 6) 1 mg/mL total protein from non-transgenic cotton plant R15. (**b**) In situ observation of *V. dahliae* with GFP of *HEWL* transgenic cotton after *V. dahliae* inoculation. Wild type: non-transgenic cotton plant R15; NT-wild type: non-treatment wide type cotton plant. Scale bars = 100 μm. All experiments were repeated at least three times.

Our previous study showed that HEWL could effectively inhibit the growth of *V. dahliae* with a bacteriostatic rate of 75.7% [29]. The in vitro fungal inhibition test showed that HEWL could effectively inhibit the growth of *V. dahliae*. Also, the protein from *HEWL* transgenic cotton could inhibit the proliferation of *V. dahliae*. The proliferation of *V. dalliae* in non-transgenic WT cotton was the same as that of the negative control (Figure 5a). We inoculated cotton plants with Vd991-GFP to observe the strength of green fluorescence in plants. After 20 days of inoculation, a strong GFP signal was observed in the stem of WT cotton, whereas a weaker GFP fluorescence was observed in the transgenic cotton plants (Figure 5b). These results indicated that the overexpression of HEWL can effectively inhibit the growth of *V. dahliae* in cotton.

## 3. Discussion

### 3.1. Fungal Inhibitory Activity of HEWL

The overexpression of PR genes and antimicrobial peptide (AMP) genes in plants can improve plant resistance to pathogens. In addition, the precise editing and knockout of target genes using CRISPR/Cas9 systems can also enhance the disease tolerance of the plant. However, very few antifungal proteins are known to impart disease resistance in crop plants. HEWL can hydrolyze the cell wall of Gram-positive bacteria to generate a bacteriostatic effect [28]. Our previous study demonstrated that HEWL can effectively inhibit growth of *V. dahliae* and *F. oxysporum* [29]. This study also confirmed that the HEWL inhibited the growth of *V. dahliae* (Figure 5a). However, the mechanism by which it inhibits fungal growth remains unclear. Düring et al. found that the α-helix at the C-terminal of T4 lysozyme has “non-enzymatic antibacterial activity”, which interferes with the cell membranes of bacteria and fungi [36]. HEWL may also have the same non-enzymatic activity. Destabilase-Lysozyme (mlDL) from *Hirudo medicalis* has a non-enzymatic antibacterial effect independent of its muramidase activity [37,38]. Therefore, the mechanism of HEWL inhibiting fungi, including enzymatic and non-enzymatic activity, needs to be further studied and determined.

### 3.2. HEWL Produces a ROS Burst in Transgenic Cotton

Pathogenic fungi produce oxidative bursts shortly after infecting plant tissues, characterized by the transient overproduction of ROS [39]. ROS usually refers to H_2_O_2_, O_2_-, OH, O_2_, and NO also belongs to ROS in a sense [40]. Oxathiapiprolin is a plant-specific pathogenic oomycete inhibitor. In a previous study, pathogen-infected *Arabidopsis thaliana* plants were treated with oxathiapiprolin to enhance H_2_O_2_ accumulation [41]. Cassava (*Manihot esculenta* Crantz) AP2/ERF transcription factor MeRAVs positively co-regulate cassava bacterial blight (CBB) resistance and stimulate innate immune responses by modulating reactive oxygen species (ROS) burst [42]. *Hcm1* transgenic cotton also generated a ROS burst after *V. dahliae* inoculation [43]. This study exhibited that after *V. dahliae* inoculation, the H_2_O_2_ and NO contents reached the peak within 1 h in *HEWL* transgenic cotton, while it reached the peak at 5 h in the WT plants (Figure 4a–c). In this study, we found that after *V. dahliae* infection, a ROS burst was triggered in cotton, which accumulated more rapidly in transgenic plants than in the wild-type plants.

### 3.3. HEWL Elicits Defense Responses against Pathogens in Cotton Plants

In the greenhouse, *HEWL* transgenic cotton plants showed stronger disease resistance and significantly decreased fungal biomass after *V. dahlia* inoculation (Figure 2a–c). In vitro experiments revealed that HEWL was able to effectively inhibit the growth of *V. dahliae*, and the protein extracted from *HEWL* transgenic cotton could also inhibit fungal growth (Figure 5a). Compared to the wild type, *HEWL* transgenic cotton plants showed strong resistance to Verticillium wilt and significantly improved disease resistance index in the field (Figure 3). These results indicated that overexpression of HEWL in cotton could effectively improve the Verticillium wilt resistance by inhibiting fungal growth. In addition, overexpression of HEWL in cotton can activate the expression of the disease-resistance PR genes (Figure 4d–j). Our results indicated that *HEWL* transgenic cotton responded to biotic stress by regulating the expression of disease resistance-related genes. Plants evoke innate immunity against pathogen challenge upon recognizing chitin in the fungal cell wall. Fujikawa et al. showed that α-1,3-glucan protected the fungal cell wall to delay innate immune defense responses [44]. Taken together, overexpression of HEWL in cotton can elicit defense responses against pathogens by hydrolyzing fungal cell walls.

### 3.4. The Strategies and Future Prospects of Controlling Cotton Diseases

There are many approaches to controlling cotton diseases, such as seed disinfection, crop management, biological control, and the breeding of resistant varieties. Environmentally friendly and ecologically safe biocontrol methods are an important strategic requirement for sustainable agricultural development. Many microorganisms have been proven to have biocontrol effects, such as *Pseudomonas* spp. and *Serratia plymutica* [45]. In addition, endophytic fungi isolated from cotton roots can also improve the resistance to Verticillium wilt [46]. However, there is no effective biological agent to control cotton disease in the market. Long-term production practice shows that biological breeding is the fundamental measure of disease control in the world. The overexpression of antimicrobial proteins and PR genes can effectively improve plant disease resistance, but the available variety for production by transgenic method must undergo the safety assessment of GM crops according to Chinese legal regulations, which is time-consuming, expensive, and complex. Recently, researchers have shown created transgene-free edited cotton lines through editing the Gh14-3-3d gene using CRISPR/Cas9 method to improve resistance against *V. dahliae* invasion [47]. Next, we will use CRISPR/Cas9 technology to develop transgene-free edited plants from transgenic lines, which is a key issue in the improvement of the breeding of transgenic disease-resistant crops.

## 4. Materials and Methods

### 4.1. Plant Materials and Growth Conditions

The cotton wild type varieties *Gossypium hirsutum* L. R15 and *Gossypium barbadense* L. Hai 7124 were provided by the Cotton Research Institute, Shanxi Academy of Agricultural Sciences. The plants were grown in a growth chamber at 25 °C under 16 h light/8 h dark conditions in Chinese Academy of Agricultural Sciences. Moreover, the medium for cotton tissue culture was previously described by Wu et al. [48], and the cotton genetic transformation materials were cultured in a light incubator (16 h light/8 h dark) at 28 °C.

### 4.2. V. dahliae Strain and Culture Conditions

*V. dahliae* strains Vd991 and Vd991-GFP (harboring GFP gene) were provided by the Institute of Plant Protection, Chinese Academy of Agricultural Sciences. The fungal strains were cultured on potato dextrose agar (PDA) medium at 25 °C for 7 days. Next, the colonies were inoculated into Czapek medium [49], and shocked at 200 rpm for 7 days at 25 °C. The conidia were counted using a hemocytometer, and their final concentration was adjusted to 5 × 10^6^ mL^−1^ with deionized water for infection, as described previously [50].

### 4.3. Cotton Transformation and Transgenic Plant Selection

The antifungal gene *HEWL* and glyphosate resistance gene *CP4-EPSPS* were synthesized by GenScript (Nanjing, China). We added a signal peptide from alpha-amylase (26 amino acids) to the N terminal of HEWL. The sequences of the genes were shown in Figure S1. We constructed the plant expression vector pBI121-35S::*CP4_EPSPS*-PetE::*HEWL*, which harbored the *HEWL* and *CP4-EPSPS* genes. The sequences of corresponding primer pairs were found in Table S1. We introduced the vector into *Agrobacterium tumefaciens* strain LBA4404 via electroporation. The *Agrobacterium* carrying vector pBI121-35S::*CP4_EPSPS*-PetE::*HEWL* was inoculated in LB liquid medium (50 mg/L Kan, 50 mg/L Rif) and cultured at 200 rpm for 48 h at 28 °C. The cell suspension was diluted with MS liquid medium to OD600 0.4 for a subsequent experiment. Hypocotyls of upland cotton R15 sterile seedlings were infected using the *Agrobacterium*-mediated method [48] and were cultured under light conditions (16 h light/8 h dark) at 28 °C in an incubator. The transgenic seedlings obtained by tissue culture were grafted to the rootstock *Gossypium barbadense* L. Hai 7124 and cultivated in the greenhouse.

### 4.4. PCR and qPCR

The genomic DNA of cotton leaf was extracted using the CTAB method [51]. Using the isolated DNA as a template, the target genes, *CP4-EPSPS* and *HEWL,* were amplified. PCR amplification was performed according to Guo et al. [52].

The total RNA of cotton tissue was extracted using the plant RNA extraction kit (Sangon Biotech, Shanghai, China). cDNA was synthesized using the reverse transcription kit EasyScript^®^ All-in-One First-Strand cDNA Synthesis SuperMix (TransGen, Beijing, China). Using cDNA as a template, qPCR was performed using TransStart^®^ Tip Green qPCR SuperMix Kit (TransGen, Beijing, China) on the ABI Prism 7500 real-time PCR platform (Applied Biosystems, Foster City, CA, USA). qPCR amplification program was as follows: 95 °C for 10 min; 95 °C for 10 s; and 60 °C for 30 s for 40 cycles of amplification. Three biological replicates and three technical replicates were performed for each sample. Each biological replicate was obtained from three plants of each line as one sample. PCR and qPCR-related gene primers were shown in Table S1.

### 4.5. Southern Blot and Western Blot

For Southern blot analysis, 30 µg gDNA from the young leaves of *HEWL* transgenic cotton and WT was extracted and digested with *Eco*RI or *Nde*I, respectively. We labelled the *CP4-EPSPS* gene as a probe via PCR with DIG Probe Synthesis Kit (Roche Applied Science, Mannheim, Germany), and the primer pairs (CP4-F3, CP4-R3) were shown in Table S1. The DNA electrophoresed on the gel was transferred to a nylon membrane. Southern blotting was performed using DIG High Prime DNA Labeling and Detection Starter Kit II (Roche Applied Science, Mannheim, Germany). The DIG-labeled DNA Molecular-Weight Marker was also obtained from Roche Applied Science.

The Western blot analysis was conducted as described by Jorgensen et al. [53]. Total plant protein was extracted from *HEWL* transgenic and WT cotton leaves [54]. After electrophoresis on 15% SDS-PAGE, the protein was transferred to a nitrocellulose membrane (20 V, 10 h) and blocked with a blocking solution at 25 °C for 2~3 h. Next, the anti-rabbit HEWL antibody (ABclonal, Wuhan, China) and goat anti-rabbit IgG-AP (Abcam, Cambridge, UK) was added. The dye was BCIP/NBT Alkaline Phosphatase reagent (Solarbio, Beijing, China).

### 4.6. Evaluation of Cotton Resistance to Verticillium Wilt

Cotton seeds were sown in nursery trays and grown normally for 1 month under greenhouse conditions. One-month-old cotton seedlings were inoculated using *V. dahliae* spore suspension using the root irrigation method [55]. Each cotton plant was treated with 20 mL conidial suspension (5 × 10^6^ conidia/mL). We treated at least 30 plants per line. The infection assay was repeated at least four times in the greenhouse. We observed the incidence of cotton and counted the disease index. Vd991-GFP was used to infect one-month-old cotton seedlings using the root irrigation method under greenhouse conditions. After 1 month, the plants showed disease symptoms. Then, the plant roots were collected and sliced for sample preparation for subsequent microscopic observation. Samples were observed using a confocal microscope (Zeiss LSM700, Jena, Germany). Each sample was taken from three treated plants of each line.

The field disease resistance was evaluated in *V. dahliae* disease-field of the Institute of Plant Protection of CAAS, Langfang, Hebei Province and the Institute of Cotton Research of CAAS, Anyang, Henan Province. The Jimian11 and 86-1varieties susceptible to Verticillium wilt were used as the susceptible control, and Zhongzhimian2 and ZhongzhimianKV3 were planted as the disease-resistant control. The experiment adopted 2 rows of planting with about 50 plants in each plot. Transgenic lines, non-transgenic WT, and the control were sown in rows with a complete block design with four replicates. The disease incidence was investigated in mid-September, and the disease index was counted.

Cotton disease index and five disease grade was calculated as described by Tong et al. [9]. Plant disease grade was scored according to the following: 0 = no fungal infections; 1 = <25% diseased leaves; 2 = 25–50% leaves showing yellow spots; 3 = 50–75% leaves showing colored spots and rolled leaf edges; 4 = >75% diseased leaves. The disease index (DI) was calculated according to the following formula: DI = [∑(disease grades × number of infected plants)/(total checked plants × 4)] × 100.

### 4.7. Trypan Blue and DAB Staining

Cotton leaves were stained with trypan blue [56]. The leaves were first placed in a decolorizing medium (absolute ethanol: acetic acid = 1:1) for 24 h at room temperature. After washing with distilled water, the leaves were soaked in trypan blue dye (10 mL lactic acid, 10 mL glycerol, 10 g phenol, and 10 mg trypan blue dissolved in 10 mL ddH_2_O), soaked for 6–8 h and decolorized with 2.5 g/mL chloral hydrate solution. We observe the stained leaves under a stereoscopic microscope (Olympus SZX7, Tokyo, Japan). DAB staining is able to detect H_2_O_2_ through observing brown polymerization product in leaves of plants [57]. Cotton leaves were stained using the Metal Enhanced DAB Substrate Kit (Solarbio, Beijing, China). Leaves were stained in DAB staining solution overnight in the dark and washed with 96% ethanol until the chlorophyll was completely decolorized. Further, the leaves were also observed with stereoscopic microscope (Olympus SZX7, Tokyo, Japan). The leaf smeared with *V. dahliae* spore suspension from each plant was treated as one sample. Each experiment was repeated at least four times.

### 4.8. Determination of H_2_O_2_, NO Content

The H_2_O_2_ content was determined using a hydrogen peroxide (H_2_O_2_) content detection kit (Solarbio, Beijing, China). Catalase (CAT) activity was measured using the Micro Catalase (CAT) Assay Kit (Solarbio, Beijing, China). NO content was determined using the Micro NO Content Assay Kit (Solarbio, Beijing, China). The average value was determined using at least four Verticillium-inoculated plants for each line.

### 4.9. Statistical Analysis

All assays were independently repeated at least three times and the presented data represent the mean ± SE. The effect of treatment was determined via analysis of variance (ANOVA), and mean values were compared with Duncan’s multiple range tests using SPSS19.0 software (IBM, New York, NY, USA).

## Data Availability

Data supporting the findings of this work are provided in the paper and its Supplementary Materials. The genetic materials supporting the findings of the current study are available from the corresponding authors upon request.

## Supplementary Materials

The following supporting information can be downloaded at: https://www.mdpi.com/article/10.3390/ijms242417164/s1.
