# Supplementary material for: Hen Egg White Lysozyme (HEWL) Confers Resistance to Verticillium Wilt in Cotton by Inhibiting the Spread of Fungus and Generating ROS Burst
Source: Int J Mol Sci. 2023 Dec 5;24(24):17164. doi: 10.3390/ijms242417164 (PMC10743298; doi:10.3390/ijms242417164)
Supplement: Supplementary file 1 [file ijms-24-17164-s001.zip › ijms-2742818-supplementary.pdf]

Hen Egg White Lysozyme (HEWL) Confers Resistance to Verticillium Wilt in Cotton by Inhibiting the Spread of Fungus and Generating ROS Burst

Wenfang Guo *, Gangqiang Li, Nan Wang, Caifeng Yang, Huakang Peng, Mengqi Wang and Dehu Liu *

Biotechnology Research Institute, Chinese Academy of Agricultural Sciences, Beijing 100081, China; lgq-1969@163.com (G.L.); wangnan@caas.cn (N.W.); ycfcaas@163.com (C.Y.); canmiki@outlook.com (H.P.); 13937133482@163.com (M.W.)

* Correspondence: guowf87@163.com (W.G.); liudehu2006@126.com (D.L.)

*npt*II:
atgattgaacaagatggattgcacgcaggttctccggccgcttgggtggagaggctattcggctatgactgggcacaacagacaatcggctgctctgatg
ccgccgtgttccggctgtcagcgcaggggcgcccggttctttttgtcaagaccgacctgtccggtgccctgaatgaactgcaggacgaggcagcgcggct
atcgtggctggccacgacgggcgttccttgcgcagctgtgctcgacgttgtcactgaagcgggaagggactggctgctattgggcgaagtgccggggc
aggatctcctgtcatctcaccttgctcctgccgagaaagtatccatcatggctgatgcaatgcggcggctgcatacgcttgatccggctacctgcccattcga
ccaccaagcgaaacatcgcatcgagcgagcacgtactcggatggaagccggtcttgtcgatcaggatgatctggacgaagagcatcaggggctcgcgc
cagccgaactgttcgccaggctcaaggcgcgcatgcccgacggcgatgatctcgtcgtgacccatggcgatgcctgcttgccgaatatcatggtggaaaa
tggccgcttttctggattcatcgactgtggccggctgggtgtggcggaccgctatcaggacatagcgttggctacccgtgatattgctgaagagcttggcg
gcgaatgggctgaccgcttcctcgtgctttacggtatcgccgctcccgattcgcagcgcatcgccttctatcgccttcttgacgagttcttctga

CP4-EPSPS:
atggcacaaattaacaacatggcacaagggatacaaacccttaatcccaattccaatttccataaaccccaagttcctaaatcttcaagttttcttgtttttggat
ctaaaaaactgaaaaattcagcaaattctatgttggttttgaaaaaagattcaatttttatgcaaaagttttgttcctttaggatttcagcatcagtggctacagc
ctgcatgcttcacggtgcaagcagccggcccgcaaccgcccgcaaatcctctggcctttccggaaccgtccgcattcccggcgacaagtcgatctcccacc
ggtccttcatgttcggcggtctcgcgagcggtgaaacgcgcatcaccggccttctggaaggcgaggacgtcatcaatacgggcaaggccatgcaggcca
tgggcgccaggatccgtaaggaaggcgacacctggatcatcgatggcgtcggcaatggcggcctcctggcgcctgaggcgccgctcgatttcggcaat
gccgccacgggctgccgcctgaccatgggcctcgtcggggtctacgatttcgacagcaccttcatcggcgacgcctcgctcacaaagcgcccgatgggcc
gcgtgttgaacccgctgcgcgaaatgggcgtgcaggtgaaatcggaagacggtgaccgtcttcccgttaccttgcgcgggccgaagacgccgacgccg
atcacctaccgcgtgccgatggcctccgcacaggtgaagtccgccgtgctgctcgccggcctcaacacgcccggcatcacgacggtcatcgagccgatca
tgacgtgcgatcatacggaaaagatgctgcagggctttggcgccaaccttaccgtcgagacggatgcggacggcgtgcgcaccatccgcctggaaggc
cgcggcaagctcaccggccaagtcatcgacgtgccgggcgacccgtcctcgacggccttcccgctggttgcggccctgcttgttccgggctccgacgtca
ccatcctcaacgtgctgatgaaccccacccgcaccggcctcatcctgacgctgcaggaaatgggcgccgacatcgaagtcatcaacctgcgccttgccgg
cggcgaagacgtggcggacctgcgcgttcgctcctccacgctgaagggcgtcacggtgccggaagaccgcgcgcctccgatgatcgacgaatatccga
ttctcgctgtcgccgccgccttcgcggaaggggcgaccgtgatgaacggtctggaagaactccgcgtcaaggaaagcgaccgcctctcggccgtcgcca
atggcctcaagctcaatggcgtggattgcgatgagggcgagacgtcgctcgtcgtgcgtggccgccctgacggcaaggggctcggcaacgcctcgggc
gccgccgtcgccacccatctcgatcaccgcatcgccatgagcttcctcgtcatgggcctcgtgtcggaaaaccctgtcacggtggacgatgccacgatgat
cgccacgagcttcccggagttcatggacctgatggccgggctgggcgcgaagatcgaactctccgatacgaaggctgcctga

HEWL:

Aaagtctttggacgatgtgagctggcagcggctatgaagcgtcacggacttgataactatcggggatacagcctgggaaactgggtgtgtgttgcaaaa
ttcgagagtaacttcaacacccaggctacaaaccgtaacaccgatgggagtaccgactacggaatcctacagatcaacagccgctggtggtgcaacgat
ggcaggaccccaggctccaggaacctgtgcaacatcccgtgctcagccctgctgagctcagacataacagcgagcgtgaactgcgcgaagaagatcgt
cagcgatggaaacggcatgagcgcgtgggtcgcctggcgcaaccgctgcaagggtaccgacgtccaggcgtggatcagaggctgccggctgtga

Figure S1. The gene sequences of *npt*II, *CP4-EPSPS*, *HEWL* in the plant expression vector pBI121-35S::*CP4_EPSPS*-PetE::*HEWL*.

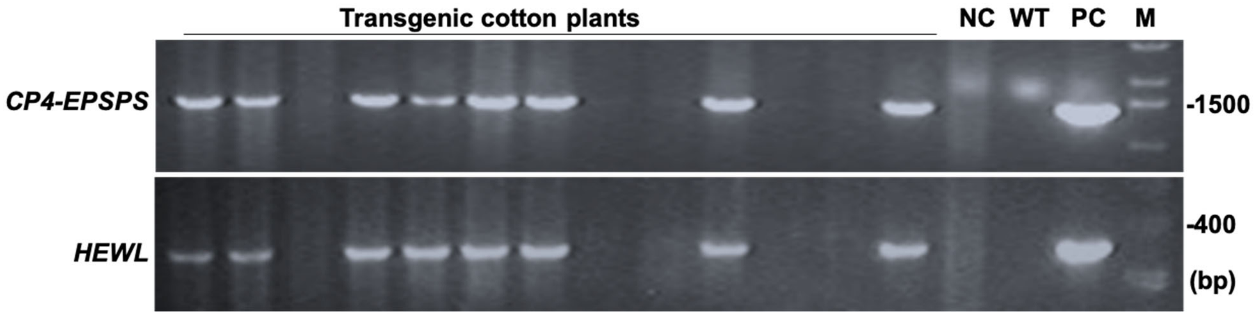

Figure S2. PCR analysis of T_0 transgenic cotton plants generation. NC: blank control; WT: wild type; PC: plasmid as positive control.

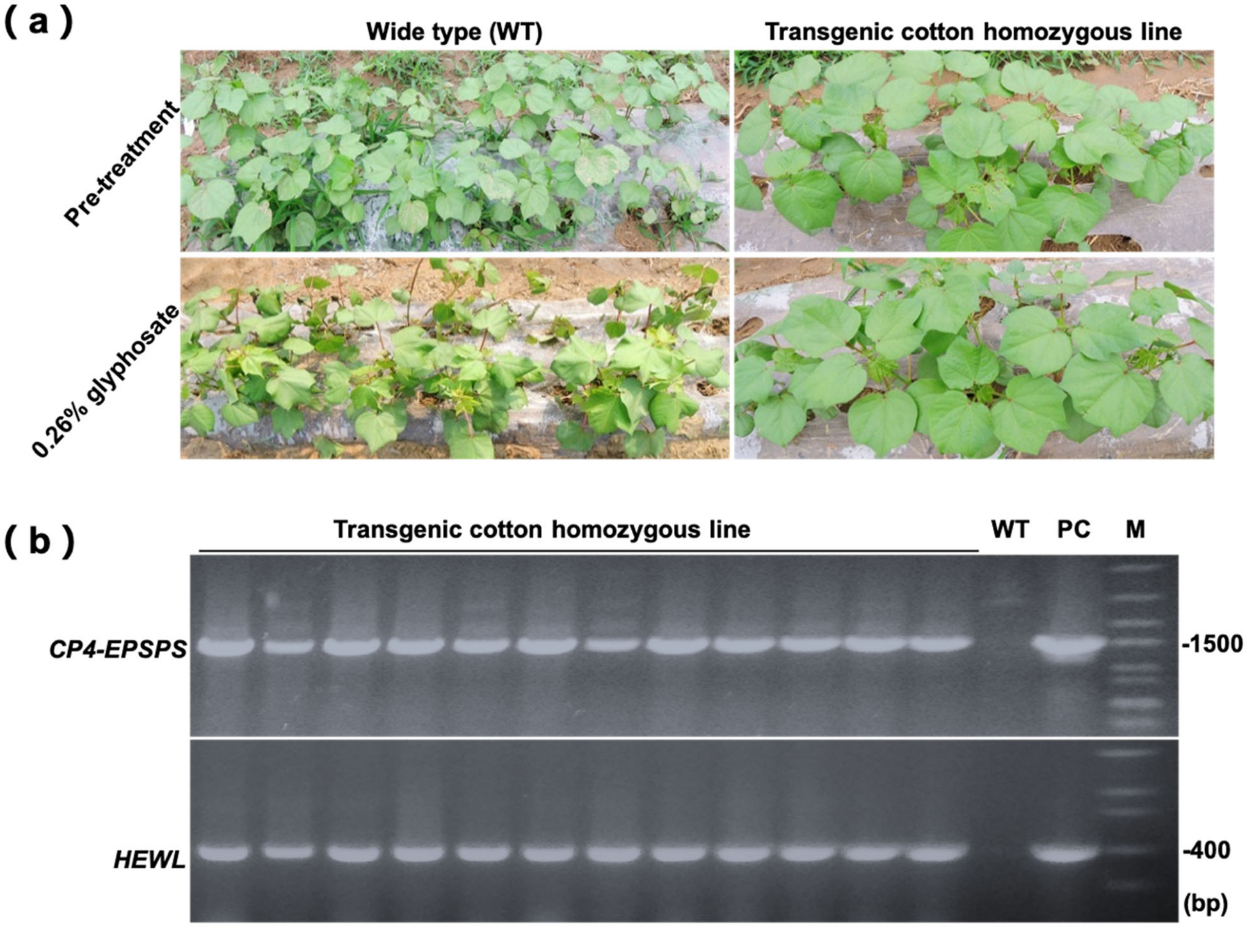

Figure S3. Identification of transgenic cotton homozygous line plants. (a) 0.26% glyphosate screening of transgenic cotton homozygous line. (b) PCR analysis of transgenic cotton homozygous line. WT: wild type; PC: plasmid as positive control.

Table S1. The primers used in this study.

**Primers**	**Sequences（5′-3′）**	**Purpose**
CP4-F1	AGGATCCAACAATGGCACAAATTAACAACATGGCACAAG	Vector construction
CP4-R1	ACTCGAGTTAGGCAGCCTTCGTATCGGAGAG	
HEWL-F1	CTCGAGATGAAAGTCTTTGGACGATGTGAGCTGG	Vector construction
HEWL-R1	GCGGCCGCTCACAGCCGGCAGCCTCTGATCCACGC	
CP4-F2	ATGGCACAAATTA ACAACATGGCACAAG	Detection of target gene
CP4-R2	TTAGGCAGCCTTCGTATCGGAGAGTTCGATC	
HEWL-F2	AAAGTCTTTGGACGATGTGAGCTGGCAGCGGCTATGAAGC	Detection of target gene
HEWL-R2	GCCGTTTCCATCGCTGACGATCTTC	
CP4-F3	CTCGTCGGGGTCTACGATTTCGACAGC	Southern blot
CP4-R3	TCAGGCAGCCTTCGTATCGGAGAGTTC	
GhActin-F	ATCCTCCGTCTTGACCTTG	qPCR (cotton reference gene)
GhActin-R	TGTCCGTCAGGCAACTCAT	
CP4-F	GTCTACGATTTCGACAGCACC	qPCR
CP4-R	ACCGTCTTCCGATTTCACC	
HEWL-F	CTATGAAGCGTCACGGACTTG	qPCR
HEWL-R	CGGTGTTACGGTTTGTAGCCT	
Vd991-F	AAAGTTTTAATGGTTCGCTAAGA	qPCR
Vd991-R	CTTGGTCATTTAGAGGAAGTAA	
GhNPR1-F	TCAGTTTAGACAAGCCCGAGAA	qPCR
GhNPR1-R	CGTATGACCCTCTTTCAGTAGCA	
GhPR1-F	TGCTGTAAATATGTGGGTTAATGAG	qPCR
GhPR1-R	GAAATTGCCTGGAGGAGAATAG	
GhNOA1-F	GAGGATGCTGAAAGACCTGCTA	qPCR
GhNOA1-R	TCTCAACTGGCTTGGGTACATG	
GhHSR203J -F	GTGATAGCTCAGGAGGGAACAT	qPCR
GhHSR203J -R	CTAACTCGGACTTGCTTCGTTG	
GhHIN1-F	GCTGATGAGACATCGGAGTTTA	qPCR
GhHIN1-R	CTACCATTCCCAGTGTTCAAAG	
GhPFN2-F	ATGATGTCGTGGCAAGCTTACG	qPCR
GhPFN2-R	TCAATAACCCTGATCGAGG	
GhPDF1.2-F	CTGTGGTAGCGGATGGTGATAAG	qPCR
GhPDF1.2-R	GTGCAGACGCATTTGCGAAGGAA	
